# The burden and clinical manifestation of hospitalized influenza among different pediatric age‐groups in the tropics

**DOI:** 10.1111/irv.12692

**Published:** 2019-10-13

**Authors:** Chia‐Yin Chong, Chee‐Fu Yung, Cherie Gan, Szu‐Tien Thio, Natalie Woon‐Hui Tan, Nancy Wen‐Sim Tee, Cui Lin, Raymond Tze‐Pin Lin, Koh‐Cheng Thoon

**Affiliations:** ^1^ Infectious Diseases Department of Paediatrics KK Women’s and Children’s Hospital Singapore; ^2^ Yong Loo Lin School of Medicine National University of Singapore Singapore; ^3^ Duke‐NUS Medical School Singapore; ^4^ Lee Kong Chian School of Medicine Nanyang Technological University Singapore; ^5^ Singhealth Residency Programme Singapore General Hospital Singapore; ^6^ Department of Laboratory Medicine National University Hospital Singapore; ^7^ National Public Health Laboratory Ministry of Health Singapore

**Keywords:** complications, disease burden, hospitalizations, influenza, pediatric

## Abstract

**Introduction:**

In tropical Singapore, influenza occurs all year‐round. This study of influenza‐confirmed hospitalized pediatric patients compared clinical characteristics and complications by age‐group and differences between influenza A and B.

**Methods:**

This was a retrospective study of pediatric inpatients from January 2013 to December 2014. Patients were grouped into: <6 months, 6 months to <5 years, 5‐ to <10‐year and ≥10 years. Complications were classified into neurologic, pulmonary, and other. We also calculated the incidence of hospitalized influenza cases per 100 000 age‐related population.

**Results:**

There were a total of 1272 patients with a median age of 37 months. The highest hospitalization rates were in the <6 months age‐group. Majority (75.2%) had no comorbidity; 25.6% had complications: neurologic 11.9%, pulmonary 9.6%, other 4.1%. Patients with other complications were older, male, and had the highest influenza B rates and the longest length of stay. Influenza A comprised 76.9% of cases and had higher complication rates especially neurologic, compared to influenza B. Influenza B patients were older and were more likely to develop other complications. The 6‐month to <5‐year‐age‐group had the highest complication rate (30.6%), especially neurologic. However, ≥10 years old had the highest other complications, ICU/ high‐dependency admissions and influenza B Victoria rates.

**Conclusions:**

Infants <6 months had the highest hospitalization rates for influenza. The 6‐month to <5‐year‐age‐group had the highest complication rate especially neurologic. Influenza A patients were younger, had higher seizure rates and complications compared to influenza B.

## 1 INTRODUCTION

In tropical Singapore, influenza is reported year‐round with two peaks between April to July and November to January.^1^, ^2^ This seasonal increase coincides with the southern and northern hemisphere winter influenza seasons, respectively. Influenza‐related hospitalization in Singapore was highest in persons ≥75 years and second highest among children <6 months of age, followed by 6‐23 months and 5‐14 years of age.^3^ In our previously published study, age <2 years and comorbidity were risk factors for complicated influenza during the influenza A (H1N1) 2009 pandemic.^4^ Other known risk factors for complicated influenza are as follows: chronic respiratory, cardiac, metabolic, renal, neurologic diseases, immunosuppression, long‐term aspirin, hemoglobinopathies, prematurity, morbid obesity, and age <2 years old.^5^, ^6^ While there are many published articles on influenza in children from temperate countries, few have described the burden of disease and complications in children in the tropics.

The purpose of our study was to determine the age‐specific incidence of laboratory‐confirmed influenza hospitalization, describe clinical characteristics of complications by age‐group and compare influenza A with B. We also plotted the monthly influenza subtypes over the 2 years. Patients were grouped into: <6 months, 6 months to <5 years, 5‐ to <10‐year, and ≥10 years.

## 2 METHODS

We conducted a retrospective study of pediatric inpatients with laboratory‐confirmed influenza at KK Women's and Children's Hospital (KKH) admitted from January 2013 to December 2014 over four consecutive influenza seasons. KKH is a tertiary hospital with ~500 pediatric and neonatal beds and admits 56.8% of pediatric admissions throughout Singapore with ~32 000 pediatric admissions per year (data from Ministry of Health, [MOH] Singapore). Patients routinely undergo testing for respiratory viral pathogens when clinically indicated. Nasal swabs were sent for immunofluorescence (IF) antigen testing for influenza A, B, respiratory syncytial virus (RSV), parainfluenza 1,2,3, adenovirus, and metapneumovirus (Direct fluorescent antibody (D^3^ Ultra DFA Respiratory Virus Screening & ID Kit; Diagnostic Hybrids) or multiplex polymerase chain reaction (PCR) testing for additional parainfluenza 4, rhinovirus, enterovirus, bocavirus, human coronavirus OC43, and 229E/NL63 viruses. (Commercial multiplex PCR kit: Seeplex RV15 ACE detection kit; Seegene). All pediatric inpatients ≤19‐year‐old influenza positive by PCR or immunofluorescence antigen were included. Subtyping of influenza isolates was performed at the National Public Health Laboratory in accordance to published protocols.^7^, ^8^, ^9^

Cases were classified as complicated and uncomplicated. Complicated cases were defined as those who had influenza‐related complications and presented with neurologic, pulmonary, or other (non‐neurologic/pulmonary) signs and symptoms. Uncomplicated cases were defined as upper respiratory tract manifestations and influenza‐unrelated illness, for example, urinary tract infection (UTI). High dependency (HD) is a unit that caters for more severely ill patients requiring closer monitoring or non‐invasive ventilation without needing ICU admission. Obesity was defined as BMI > 25 kg/m^2^. Immunosuppression was defined as non‐malignancy related immunosuppressive therapy, for example, steroids, biological modifying agents. Pneumonia was defined as fever and cough with chest X‐ray (CXR) changes of lobar/broncho‐pneumonia or focal infiltrates. Positive bacterial tests were either culture‐ positive from any site or positive mycoplasma PCR or serology (particle agglutination IgG antibody ≥320 single titer). C‐reactive protein was the result on admission. Criteria for treating influenza with oseltamivir were as follows: age <2 years or any high‐risk factor for complicated disease within 3 days of onset, or severe disease based on clinician assessment independent of duration since onset.

Statistical analysis was performed using the spss 19.0 statistical software program. Student's *t* test or one‐way ANOVA were used to compare continuous data; chi‐square test or Fisher's exact test was used to compare categorical data, using *P* < .05 as statistical significance. This study was approved by the internal review board for waiver of consent.

Incidence of influenza hospitalization was derived using 56.8% market share for admissions in children and calculated as per 100 000 population based on the number of children in that age‐group (information from MOH and Singapore government statistics).

$\text{Hospitalized influenza cases per}\;10^{5}\;\text{population per agegroup}=\text{No. of influenza cases in agegroup}\times \frac{100}{56.8}\times \frac{100,000}{\text{no. of children in agegroup}}$

## 3 RESULTS

There were a total of 1272 influenza‐positive patients admitted with a median age of 37 months (IQR 13‐76 months); males constituted 56.5%. Majority were positive for influenza by IF (96.5%) compared to PCR (3.5%). The median length of stay (LOS) was 3 days (IQR 2‐4 days). C‐reactive protein and chest X‐ray were performed in 28.8% and 31.1% of patients, respectively.

**Figure 1:**
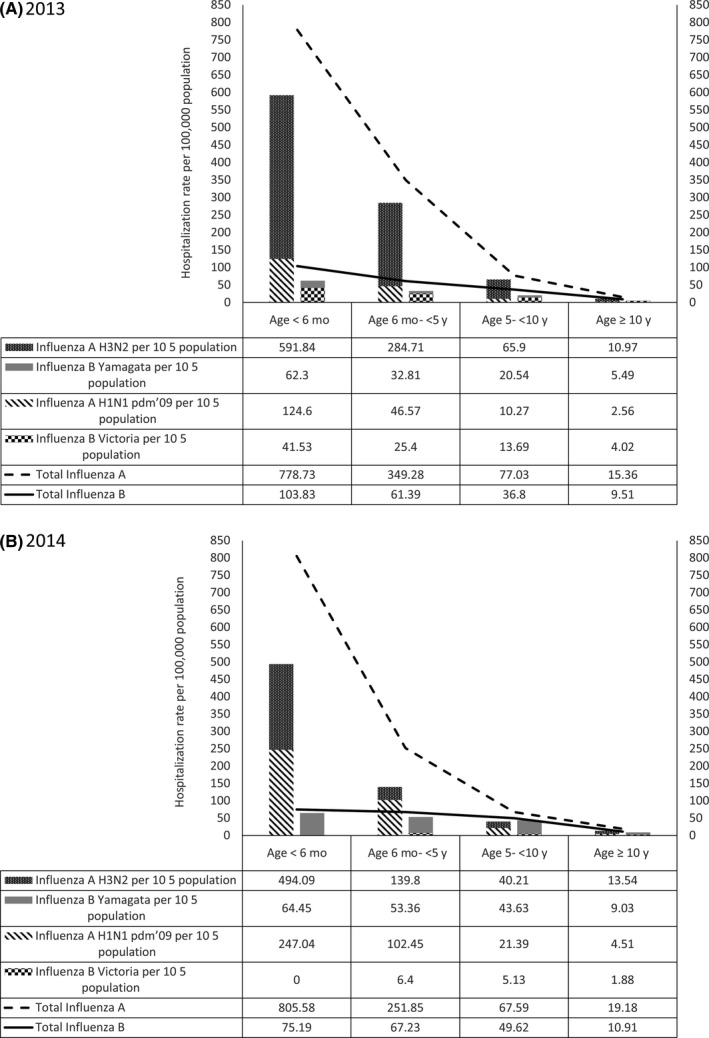
Influenza hospitalization rate per 100 000 population by subtype and age group

**Figure 2:**
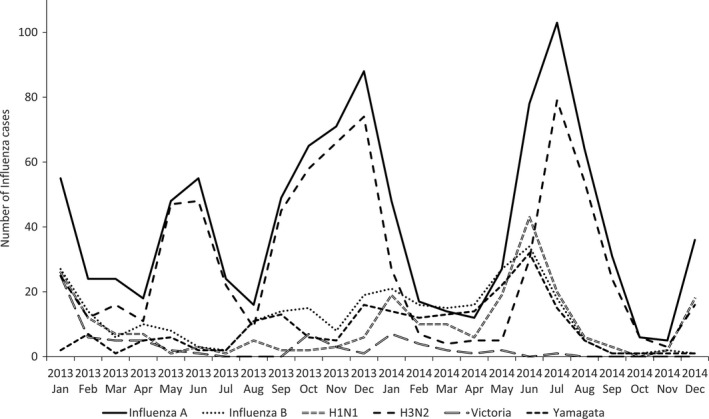
Influenza subtypes by month and year

Figure 1 shows the burden of influenza hospitalizations by year, age‐groups, and subtypes/lineages (excluding unknown subtypes/lineages). Incidence of hospitalized influenza was highest for <6 months, followed by 6‐month to <5‐year‐age‐group and lowest in ≥10‐year‐age‐group. Figure 2 shows the monthly incidence of influenza A, B, and subtypes/lineages, excluding unknown subtypes/lineages. Influenza cases peaked in June, December 2013, and July 2014. The seasonal peaks were mainly driven by influenza A H3N2. The predominant lineage for influenza B was Yamagata with peaks in September, December 2013, and June 2014. Co‐circulation of all four virus subtypes/lineages was seen throughout both years except August‐December 2014 when influenza B Victoria was undetected.

Table 1 shows the comparison between the age‐groups and whole cohort with significance testing only between age‐groups. Of those <6 months old, 25.1% were <6 weeks and 78.4% were ≤3 months. Viral coinfections occurred in 13 patients (1.02%) due to: 7 RSV, 3 parainfluenza, one each for metapneumovirus, cytomegalovirus, and varicella

**Table 1:** Comparison of characteristics and complications by age‐groups

Characteristic	Overall (n = 1272; %)	Age <6 mo (n = 167; %)	Age 6 mo‐<5 y (n = 687; %)	Age 5 to <10 y (n = 270; %)	Age ≥10 y (n = 148; %)	*P* value
Male gender	719 (56.5)	95 ( 56.9)	381 (55.5)	165 (61.1)	78 (52.7)	.48
Race						
Chinese	646 (50.8)	92 (55.1)	353 (51.4)	136 (50.3)	65 (43.9)	.36
Malay	384 (30.2)	39 (23.4)	212 (30.9)	81 (30.0)	52 (35.1)	
Indian	149 (11.7)	25 (15.0)	67 (9.8)	36 (13.3)	21 (14.2)	
Other	93 (7.3)	11 (6.6)	55 (8.0)	17 (6.3)	10 (6.8)	
Nosocomial	31 (2.4)	3 (1.8)	15 (2.2)	7 ( 2.6)	6 (4.1)	.69
Symptoms						
Fever duration (d) ±SD	2.89 ± 3.08	1.50 ± 0.96	3.22 ± 3.43	3.13 ± 3.27	2.48 ± 1.80	<.001
Cough	1011 (79.4)	111 (66.5)	569 (82.8)	219 (81.1)	112 (75.7)	<.001
Cough duration (d) ±SD	5.06 ± 8.92	2.87 ± 4.41	5.64 ± 9.40	4.88 ± 9.06	4.66 ± 9.18	.027
Runny nose	803 (63.1)	65 (38.9)	473 (68.9)	177 (65.6)	88 (59.5)	<.001
Vomiting	373 (29.3)	29 (17.4)	209 (30.4)	98 (36.3)	37 (25.0)	.001
Diarrhea	132 (10.4)	9 (5.4)	93 (13.5)	20 (7.4)	10 (6.8)	.002
Wheeze	46 (3.6)	2 (1.2)	29 (4.2)	9 (3.3)	6 (4.1)	.042
Influenza‐like illness	993 (78.1)	105 (62.9)	562 (81.8)	215 (79.6)	111 (75.0)	<.001
Shortness of breath	111 (8.7)	5 (3.0)	70 (10.2)	19 (7.0)	17 (11.5)	.006
Seizures on presentation	175 (13.7)	0	130 (18.9)	28 (10.4)	17 (11.5)	<.001
Influenza A	978 (76.9)	150(90.4)	566 (82.4)	169 (62.6)	93 (62.8)	<.001
Influenza B	294 (23.1)	16 (9.6)	121 (17.6)	101 (37.4)	55 (37.2)	
Influenza Subtype						<.001
A (H1N1) pdm09	231 (18.2)	35 (21.)	140 (20.4)	37 (13.7)	19 (12.8)	
A (H3N2)	693 (54.5)	103 (62)	400 (58.2)	124 (45.9)	66 (44.6)	
A untypeable	54 (4.2)	12 (7.2)	26 (3.8)	8 (3.0)	8 (5.4)	
B Victoria	72 (5.7)	4 (2.4)	30 (4.4)	22( 8.1)	16 (10.8)	
B Yamagata	207 (16.3)	11 (6.6)	81 (11.8)	75 (27.8)	39 (26.4)	
B untypeable	15 (11.8)	1 (0.6)	10 (1.5)	4 (1.5)	0	
Comorbidity^a^						
Comorbidity	316 (24.8)	18 (10.8)	140 (20.4)	81 ( 30.0)	77 (52.0)	<.001
1 comorbidity	218 (17.1)	15 (9.0)	98 (14.3)	60 (22.2)	45 (30.4)	
≥2 comorbidity	98 (7.7)	3 (1.8)	42 (6.1)	21 ( 7.8)	32 (21.6)	
Prior seizure history	73 (5.7)	0	35 (5.1)	25 (9.3)	13 (8.8)	<.001
Asthma	105 (8.3)	1 (0.6)	39 (5.7)	35 (13.0)	30 (20.3)	<.001
Neurologic disease	81 (6.4)	0	36 (5.2)	24 ( 8.9)	21 (14.2)	<.001
Developmental delay	61 (4.8)	1 (0.6)	29 (4.2)	14 (5.2)	17 (11.5)	<.001
Malignancy	10 ( 0.8)	0	3 (0.4)	2 (0.7)	5 (3.4)	.004
Immunosuppression	6 (0.5)	0	0	3 (1.1)	3 (2.0)	.007
Gastroesophageal reflux	18 (1.3)	0	11 (1.6)	3 (1.1)	4 (2.7)	.021
Obesity	38 (3.0)	1 (0.6)	10 (1.5)	6 (2.2)	21 (14.2)	<.001
Viral coinfection	13 (1.0)	1 (1.5)	12 (1.7)	0	0	.056
Pneumonia	56 (4.4)	1 (0.6)	34 (4.9)	17 (6.3)	4 (2.7)	.045
ICU or HD	56 (4.4)	4 (2.4)	22 (3.2)	11 ( 4.1)	19 (12.8)	<.001
Death/sequelae	4 (0.3)	0	1 (0.1)	2 (0.7)	1 (0.7)	.447
Complication	325 (25.6)	10 (6.0)	210 (30.6)	66 (24.4)	39 (26.4)	<.001
Neurologic	151 (11.9)	1 (0.6)	106 (15.4)	30 (11.1)	14 (9.5)	
Pulmonary	122 (9.6)	7 ( 4.2)	87 (12.7)	18 (6.7)	10 (6.8)	
Other	52 (4.1)	2 ( 1.2)	17 (2.5)	18 (6.7)	15 (10.1)	
Nil/unrelated	947 (74.4)	157(94.0)	477 (69.4)	204 (75.6)	109 (73.6)	
C‐reactive protein (mg/L) ±SD	17.86 ± 34.81	4.14 ± 8.26	25.97 ± 44.72	23.71 ± 40.26	18.78 ± 24.03	<.001
Oseltamivir use	25 (2.0)	3 (1.8)	11 (1.6)	5 ( 1.9)	6 (4.1)	.391
Any antibiotic use	361 (28.4)	97 (58.1)	159 (2.3)	63 (23.3)	42 (28.4)	<.001
Length of stay (d) ±SD	7.15 ± 59.6	3.29 ± 3.88	8.55 ± 77.12	4.19 ± 14.63	10.53 ± 50.33	.52

Abbreviation: SD, standard deviation.

a Besides comorbidities listed in Table 2, the remaining comorbidities were as follows: prematurity 4.9%, cardiac disease 4.6%, congenital malformation 3.5%, obesity 3%, chromosomal defects 1.2%, lung disease 1%, Kawasaki disease on long‐term aspirin 0.2%, rheumatological condition 0.1%, diabetes mellitus 0.1%. Premature babies had a mean gestational age of 32.8 wk (IQR 31‐35 wk) with a mean birth weight of 1.87 kg (IQR 1.29‐2.25 kg).

The 6‐month to <5‐year‐age‐group (54.0%) and ≥10‐year‐age‐group (11.6%) constituted the largest and smallest proportion, respectively. The 5‐ to <10‐year and ≥10‐year‐age‐groups had the highest rates of influenza B, while <6‐month‐age‐group had the highest rates of influenza A. The <6‐month‐age‐group had the influenza‐related complication rates but the highest bacterial infection rates and antibiotic usage. The 6‐month to <5‐year‐age‐group had the highest seizure rates, cough duration, CRP, and complication rates. The 5‐ to <10‐year‐age‐group had the highest influenza B Yamagata rates and prior seizure history. The ≥10‐year‐age‐group had the highest rates of comorbidity, other complications, ICU/HD admissions, and mean LOS.

Influenza‐unrelated illnesses occurred in 33 patients (2.6%), for example, UTI (n = 12), allergic reactions (n = 8), salmonella gastroenteritis (n = 2), cellulitis (n = 2) mesenteric adenitis (n = 2), eye infections (n = 2), one each for intestinal ileus, appendicitis, eczema flare, *H pylori*‐related gastroesophageal bleed, hematuria. UTI was mainly from infants <6 months old and allergic reactions in patients ≥10 years old.

Influenza A constituted 76.9% of cases, from H3N2 (54.5%), H1N1 (18.2%), and untypeable (4.2%) subtypes. (Table 2 excluded untypeable) Influenza B constituted 23.1% of cases from Yamagata (16.3%), Victoria (5.7%), untypeable (1.2%) subtypes. One patient had both influenza A H1N1‐2009 and B Yamagata. Influenza A H3N2 dominated in <6 months old, but this decreased with increasing age. The ≥5‐year‐old‐age‐groups had the highest influenza B rates (35.9%‐37.2%). Influenza A patients were younger, had higher complication rates and neurologic complications compared to influenza B (Table 3). In contrast, influenza B patients were older, had developmental delay, higher diarrhea rates, and other complications.

**Table 2:** Comparison of clinical characteristics between Influenza A and B

Clinical Characteristic	Influenza A (n = 978; %)	Influenza B (n = 294; (%)	*P* value	Odds ratio (95% CI)	Influenza A H1N1‐pdm09 (n = 231; %)	Influenza A H3N2 (n = 693; %)	Influenza B Victoria (n = 72; %)	Influenza B Yamagata (n = 207; %)
Mean age (mo) ±SD	45.9 ± 46.2	70.2 ± 50.2	<.001		42.8 ± 43.1	46.8 ± 46.4	74.3 ± 53.1	70.7 ± 49.8
Male gender	550 (56.2)	169 (57.5)	.706		134 (58.0)	384 (55.4)	41 (56.9)	119 (57.5)
Year								
2013	537 (54.9)	137 (46.6)	.012	1.40 (1.07‐1.81)	75 (34.5)	433 (62.5)	55 (76.4)	76 (36.7)
2014	441 (45.1)	157 (53.4)			156 (67.5)	260 (37.5)	17 (23.6)	131 (63.3)
Developmental delay	39 (4.0)	22 (7.5)	.014	1.95 (1.14‐3.34)	7 (3.0)	30 (4.3)	4 (5.6)	17 (8.2)
Febrile seizure	109 (11.1)	10 (3.4)	<.001	3.56 (1.84‐6.90)	16 (6.9)	93 (13.4)	2 (2.8)	8 (3.9)
Any seizure	154 (15.7)	21 (7.1)	<.001	2.43 (1.51‐3.92)	34 (14.7)	117 (16.9)	4 (5.6)	17 (8.2)
Diarrhea	87 (8.9)	45 (15.3)	.002	0.54 (0.37‐0.80)	29 (12.6)	52 (7.5)	13 (18.1)	30 (14.5)
Pneumonia	40 (4.1)	16 (5.4)	.322		14 (6.1)	20 (2.9)	7 (9.7)	9 (4.3)
Comorbidity	234 (23.9)	82 (27.9)	.168		41 (17.7)	139 (20.1)	16 (22.2)	55 (26.6)
ICU/HD admission	43 ( 4.4)	13 (4.4)	.985		10 (4.3)	24 (3.5)	2 (2.8)	11 (5.3)
Complicated influenza	267 (27.3)	58 (19.7)	.009	1.53 (1.11‐2.10)	43 (18.6)	26 (29.7)	15 (20.8)	40 (19.3)
Complication type			<.001					
Neurologic	135 (13.8)	16 (5.4)			23 (10.0)	110 (15.9)	3 (0.3)	13 (6.3)
Pulmonary	99 (10.1)	23 (7.8)			17 (7.4)	72 (10.4)	7 (9.7)	13 (6.3)
Other	33 (3.4)	19 (6.5)			3 (1.3)	24 (3.5)	5 (6.9)	14 (6.8)
Nil	711 (72.7)	236 (80.3)			188 (81.4)	487 (70.3)	57 (79.2)	167 (80.7)

Abbreviation: SD, standard deviation.

**Table 3:** Type of complications by Influenza subtype/lineage

Type of Complications	Number (n = 325; % of complications)	Influenza A H1N1‐pdm09 (n = 43; %)	Influenza A H3N2 (n = 205; %)	Influenza B Victoria (n = 15; %)	Influenza B Yamagata (n = 40; %)	Influenza A‐unknown (n = 19; %)	Influenza B‐unknown (n = 3; %)
Neurologic	151 (46.5)	23 (53.5)	110 (53.7)	3 (20.0)	13 (32.5)	2 (10.5)	0
Febrile/provoked seizure	121 (37.2)	16 (37.2)	95 (46.3)	2 (13.3)	8 (20.0)	0	0
Status epilepticus/other seizure	17 (5.2)	3 (7.0)	10 (4.9)	0	3 (7.5)	1 (5.3)	0
Encephalitis	6 (1.8)	2 (4.7)	2 (1.0)	1 (6.7)	0	1 (5.3)	0
Encephalopathy	4 (1.2)	0	3 (1.5)	0	1 (2.5)	0	0
Aseptic meningitis	2 (0.6)	1 (2.3)	0	0	1 (2.5)	0	0
Myelitis	1 (0.3)	1 (2.3)	0	0	0	0	0
Pulmonary	122 (37.5)	17 (39.5)	72 (35.1)	7 (46.7)	13 (32.5)	10 (52.6)	3 (100.0)
Pneumonia	26 (8.0)	6 (14.0)	11 (5.4)	2 (13.3)	3 (7.5)	3 (15.8)	1 (33.3)
Bronchiolitis	25 (7.7)	3 (7.0)	16 (7.8)	0	1 (2.5)	5 (26.3)	0
Croup	24 (7.4)	3 (7.0)	15 (7.3)	2 (13.3)	4 (10.0)	0	0
Asthma exacerbation	19 (5.8)	2 (4.7)	13 (6.3)	0	3 (7.5)	1 (5.3)	0
Bronchitis	12 (3.7)	1 (2.3)	7 (3.4)	1 (6.7)	1 (2.5)	1 (5.3)	1 (33.3)
Otitis media	8 (2.5)	0	6 (2.9)	1 (6.7)	0	0	1 (33.3)
Respiratory failure/ARDS	4 (1.2)	2 (4.7)	1 (0.5)	0	1 (2.5)	0	0
Sinusitis	2 (0.6)	0	1 (0.5)	1 (6.7)	0	0	0
Other pulmonary	2 (0.6)	0	2 (1.0)	0	0	0	0
Other (non‐neurologic, non‐pulmonary)	52 (16.0)	3 (7.0)	23 (11.2)	5 (33.3)	14 (35.0)	7 (36.8)	0
Myositis	11 (3.3)	0	4 (2.0)	1 (6.7)	6 (15.0)	0	0
Multi‐organ involvement	10 (2.8)^a^	1 (2.3)	5 (2.4)	0	3 (7.5)	1 (5.3)	0
Shock/pre‐shock	10 (3.3)	1 (2.3)	5 (2.4)	0	2 (5.0)	2 (10.6)	0
Acute kidney injury (AKI)	4 (1.2)	1 (2.3)	1 (0.5)	1 (6.7)	1 (2.5)	0	0
Orbital/preseptal cellulitis	4 (1.2)	0	0	1 (6.7)	0	3 (15.8)	0
Vasovagal syncope	3 (0.9)	0	2 (1.0)	1 (6.7)	0	0	0
Other	10 (2.8)^b^	0	6 (2.9)	1 (6.7)	2 (5.0)	1 (5.3)	0

a ≥2 organs, 1 each: Seizure and pneumonia; pneumonia and gastrointestinal bleed; nephrotic syndrome and asthma; shock, seizures and pneumonia; shock, Kawasaki disease and acute kidney injury (AKI); AKI and liver failure; AKI and gastrointestinal bleed; shock and hemolytic‐uremic syndrome; seizure, pneumonia, AKI and transaminitis.

b Others: Hemolytic anemia 2; mallory weiss tear 2; nephrotic syndrome exacerbation 2; 1 each: Atrial ectopics, transient synovitis, hypoglycemia with dehydration, Kawasaki disease.

Complicated influenza cases constituted 25.6% (n = 325) of which the vast majority 70.8% had no underlying comorbidity. Fifty‐six (4.4%) cases were admitted ICU or HD. Table 3 lists the type of complications. Neurologic complications were the most frequent (46.5%) followed by pulmonary (37.5%) and other (16.0%). The seven complicated cases with confirmed bacterial coinfection were as follows: two pneumococcal bacteremia, two *S pyogenes* bacteremia, one each for *P aeuginosa* pneumonia, *Mycoplasma* pneumonia, and campylobacter gastroenteritis (admitted for febrile seizure).

Overall, oseltamivir (OSV) usage was 2.0%, especially higher in complicated cases (3.6% vs 1.3%, *P* = .007), ICU/HD admissions (19.6% vs 1.2%, *P* < .001), underlying comorbidity (4.5% vs 1.3%, *P* = .002), cardiac disease (10.7% vs 1.6%, *P* < .001), and malignancy (30% vs 1.7%, *P* = .001). In contrast, any antibiotic usage was 28.4%.

Comparing patients with other complications against neurologic and pulmonary complications, they had the highest ICU/HD admissions (34.6% vs 12.6%, 10.7%, respectively, *P* < .001), highest influenza B rates (36.5% vs 10.6%, 18.9%, respectively, *P* < .001) and oldest age (mean 90.3 vs 50.6, 42.1 months respectively, *P* < .001). Pulmonary complications compared to neurologic and other complications had highest ILI rates (91.8% vs 68.2%, 76.9% respectively, *P* < .001), longest cough duration (6.5 days vs 3.2, 3.7 days, respectively, *P* = .010). LOS was not significantly different between the type of complications.

The mortality rate in our cohort was 0.2%. The three deaths were as follows: Acute necrotizing encephalitis, invasive pneumococcal disease, decompensated liver failure in a biliary atresia patient post‐ Kasai procedure. One cerebral palsy patient with no prior seizures developed sequelae of epilepsy.

## 4 DISCUSSION

The main findings from our study were that the highest burden of influenza hospitalizations was in the <6‐month‐age‐group, followed by the 6‐month to <5‐year‐age‐group. Majority (75.2%) of pediatric inpatients had no comorbidity and 25.6% had complications: neurologic 11.9%, pulmonary 9.6%, and other 4.1%. The 6‐month to <5‐year‐age‐group suffered the highest complication rate (30.6%). Influenza A patients were younger, had higher seizure rates and complications compared to influenza B.

In our study, the highest hospitalization rate occurred in the <6‐month‐age‐group. This is similar to Hong Kong but different from temperate countries where the highest rates occurred in the 6‐ to 23‐month‐age‐group. 10, 11, 12 Patients who were ≤6 weeks or <3 months old were likely admitted for neonatal or infantile pyrexia workup, this can explain the high UTI rates and antibiotic usage in this age‐group. At the average rate of 881.68 per 10^5^ population for <6 months old, it is higher than temperate countries.^3^, ^13^ This could be due to all year‐round influenza with bimodal peaks, similar to Malaysia.^14^ Studies have shown that maternal influenza vaccination can prevent influenza in infants; therefore, pregnant women should receive influenza vaccination to protect their infants.^15^, ^16^

The age‐group 6 month to <5 years was the largest cohort (54%) with the second‐highest hospitalization rate and the highest complication rate (30.6%) especially neurologic, similar to another study.^17^ In other studies, the age‐group 6‐35 months or the lowest age‐groups had the highest incidence of complications.^11^, ^18^, ^19^

However, the ≥10‐year‐old‐age‐group had the highest other complications, ICU/high‐dependency admissions, and influenza B Victoria rates. This is unlike other studies which had higher ICU admissions in the youngest age‐groups <12 months.^20^ In addition, the ≥10‐year‐old‐age‐group had the highest LOS likely from underlying comorbidity especially ≥2 comorbidities. One previous study showed that children ≥10 years old with influenza B had the greatest odds of ICU admission if previously healthy.^21^ This age‐group also had the highest influenza B Victoria rates, implying that they should receive the quadrivalent vaccine to reduce the chances of a B‐mismatched season.^22^

Our comorbidity rate is lower than other published rates of 51%‐63%.^11^, ^12^, ^21^ Among those with complicated disease, 34.2% had comorbidity. Our lower comorbidity rate may be related to the health access seeking behavior of parents of previously well children who tend to come to our hospital's emergency department (ED) after office‐hours rather than go to the primary care practitioners. If a rapid diagnostic test was offered at the ED, perhaps some of the uncomplicated influenza patients need not have been hospitalized. Targeted testing has been shown to reduce hospital admissions in children and antibiotic usage.^23^ Regardless, the absence of comorbidity in the majority (75.2%) of our cohort lends support to the use of a universal influenza vaccination in all children regardless of whether they have underlying diseases.

The most common complications were neurologic (46.5%) followed by pulmonary (37.5%). In contrast, other studies have reported pulmonary complications as the most frequent complication especially pneumonia and asthma exacerbation.^11^, ^16^ This could be related to our admission criteria to admit all febrile seizures under 18 months old (only 3.3% of febrile seizures <18 months) and afebrile seizures under 12 months old. Of the neurologic complications (n = 151), febrile seizures constituted 80% and status epilepticus 11.3%, encephalitis, encephalopathy, aseptic meningitis, myelitis 8.6%. Acute seizures contributed 42.4% of neurological complications, similar to other studies with rates of 55%‐78%.^20^, ^24^ In two published studies, encephalitis, encephalopathy, and febrile seizures constituted 0.6%‐ 4.4% of influenza admissions, whereas these constituted 10.3% of our admissions.^16^, ^20^ In a study of influenza vaccine uptake, only 51% and 46% of physicians recognized epilepsy and intellectual disability as high‐risk conditions.^25^ Physicians should routinely offer influenza vaccination to patients with a prior seizure disorder, neurologic conditions or developmental delay.^26^, ^27^

In our cohort, influenza A comprised 76.9% and had higher complication rates especially neurologic. Influenza B contributed to 23.1% of all admissions in 2013‐2014; this is similar to the 19.3% rate for Singapore in 2007‐2012.^26^ The older age‐groups ≥5 years old had higher proportions of influenza B compared to the younger age‐groups. This is similar to previous publications which showed that those aged ≥5 years were more likely to have influenza B but different from Hong Kong where the 2‐ to 4‐year‐age‐group had the highest influenza B hospitalization rates.^10^, ^21^, ^27^ In 2013‐2014, the quadrivalent influenza vaccine was not yet available; the trivalent vaccine would not have covered 5.7% of all strains and up to 10.8% of strains in those ≥10 years. Comparing the two subtypes of influenza A, H3N2 had higher rates of febrile seizures, bacterial pneumonia and lower rates of diarrhea compared to H1N1 pdm2009 (*P* = .008, .03, .02, respectively). There was no difference in clinical characteristics or severity between the two influenza B lineages, similar to a study from Guangzhou, China.^28^ In terms of seasonality, influenza A peaks were mainly driven by H3N2 which were more intense and earlier except in mid‐2014, when H1N1 pdm09 and influenza B peaked in June. This is similar to the H3N2 patterns in Shenzhen and Hong Kong in 2012‐2013.^29^ The predominant lineage for influenza B was Yamagata in both years, similar to that of Malaysia.^30^

One limitation of our study was that IF was the main mode of testing, which could lead to an underestimate of the true incidence of influenza hospitalization as PCR is a more sensitive method of testing. However, more severe cases could have been included in this study due to the higher viral burden in respiratory secretions detectable by IF rather than PCR. Obesity was likely under‐reported as only 55% of patients had their height taken, thereby leading to an omission of BMI used to determine obesity status. Another limitation was the lack of information of prior influenza vaccination; however, uptake for influenza vaccination in children has generally been poor. Although influenza vaccination is recommended for children under 5 years of age or those with underlying high‐risk factors, it is not in the national immunization schedule for children and therefore not routinely offered. As this was a hospital‐based study, the observations may have been confounded by the admitting threshold with younger aged children more likely to be admitted. Prior influenza infections would not have been detected as influenza serological testing was not available. It is possible that older children may have had partial immunity to influenza A, not develop severe influenza A and instead be hospitalized for influenza B.

## 5 CONCLUSIONS

Infants <6 months had the highest hospitalization rates for influenza. The 6‐month to <5‐year‐age‐group had the highest complication rate especially neurologic. Influenza A patients were younger, had higher seizure rates and complications compared to influenza B.
